# The JACS prospective cohort study of newly diagnosed women with breast cancer investigating joint and muscle pain, aches, and stiffness: pain and quality of life after primary surgery and before adjuvant treatment

**DOI:** 10.1186/1471-2407-14-467

**Published:** 2014-06-25

**Authors:** Deborah Fenlon, Cassandra Powers, Peter Simmonds, Joanne Clough, Julia Addington-Hall

**Affiliations:** 1 University of Southampton, Faculty of Health Sciences, Southampton SO17 1BJ, UK; 2 University of Southampton, Faculty of Medicine, Southampton General Hospital, Southampton SO17 1BJ, UK; 3 University Hospitals Southampton NHS Foundation Trust, Southampton SO16 6YD, UK

**Keywords:** Breast cancer, Arthralgia, Aromatase inhibitors, Cohort study

## Abstract

**Background:**

Breast cancer affects one in eight UK women during their lifetime: many of these women now receive adjuvant chemotherapy and hormone therapy. Joint and muscle pains, aches, and stiffness are common but the natural history, aetiology and impact of these symptoms are unknown. A cohort study of newly diagnosed women with primary breast cancer was established to explore this. In this paper we present study methods and sample characteristics, describe participants’ experience of musculoskeletal pain at baseline interview, and explore its impact on quality of life.

**Methods:**

Women with non-metastatic breast cancer were recruited following primary surgery into a multi-centre cohort study. They received questionnaires by post five times (baseline, 3, 6 , 9 and 12 months) to investigate prevalence, severity, location and correlates of musculoskeletal pain, and impact on quality-of-life. Pain was measured by the Nordic musculoskeletal questionnaire, the Brief Pain Inventory, and MSK-specific questions, and quality of life by the SF-36 and FACIT scales.

**Results:**

543 women (mean age 57 years, range 28–87, 64% postmenopausal) were recruited following surgery for primary breast cancer from breast cancer clinics in eight hospitals. Fifteen per cent of the eligible cohort was missed; 28% declined to participate. Joint or muscle aches, pains or stiffness were reported by 69% women with 28% specifically reporting joint pain/aches/stiffness. Quality of life, as measured by the FACT-B and adjusted for age, depression, surgery and analgesic use, is significantly worse in all domains in those with musculoskeletal problems than those without.

**Conclusions:**

Our findings highlights the importance of a better understanding of these symptoms and their impact on the lives of women with primary breast cancer so that healthcare professionals are better equipped to support patients and to provide accurate information to inform treatment decisions. Further papers from this study will address these issues.

## Background

Breast cancer affects one in eight women during their lifetime in the United Kingdom [1] and almost two thirds of newly diagnosed women are now likely to survive for at least 20 years [2]. This increase in cancer survivorship is largely due to adjuvant chemotherapy and hormone therapy [3]. Musculoskeletal pain and stiffness are common complaints following primary breast cancer treatment [4], but there is little information on the natural history of these symptoms and their impact on women’s lives over time. Aromatase inhibitors (AIs), such as anastrozole, letrozole and exemestane, are the recommended hormone treatment for postmenopausal women as they have been shown to be more effective in preventing recurrence than tamoxifen alone [5]; however, recent clinical reports and small cohort studies have reported that up to 74% of patients receiving adjuvant-AI therapy develop pain or stiffness in their joints [4,6-10]. This non-arthritis pain/stiffness, or arthralgia, is common in postmenopausal women [11] but a cross-sectional survey of more than 500 subjects conducted by this group demonstrated that women with breast cancer had significantly more musculoskeletal pain than women of a similar age attending breast screening clinic (62% of breast cancer patients vs 53% of healthy controls, p = 0.023) [12]. A recent cohort study supports this finding: 91 women on AIs were compared to a group of postmenopausal women without breast cancer (n = 177), with women on AIs having more severe arthralgia at six weeks than women without breast cancer [13].

The causes of joint and muscle aches/pains/ stiffness in women with breast cancer are unknown; however, oestrogen deprivation has been suggested as one possible cause [9,14,15]. Although oestrogen has no known effect on articular structures that would protect against musculoskeletal pain, it is known to influence the inflammation pathway and neural processing of nociceptive input [16,17]. Women treated with AIs have an associated decline in bone mineral density as a result of low oestrogen levels, which may further exacerbate musculoskeletal symptoms [18]. There appears to be a relationship between a shorter time since last menstrual period and the development of joint pain and stiffness (arthralgia) after primary breast cancer treatment which supports this hypothesis [9,19,20]. A recent cohort study, however, observed that the menopause-like symptoms experienced by women on AIs were independent of joint pain development suggesting that reported musculoskeletal pain is not due to oestrogen alone [21]. There is also evidence supporting the development of musculoskeletal pain following chemotherapy: 87% of a cohort of Canadian women (n = 82) reported pain as a side effect of taxane chemotherapy [22-24]. This has not been examined in large, prospective cohorts nor have the compound effects of hormone therapy and chemotherapy been explored. The lack of robust evidence on which adjuvant treatments are associated with musculoskeletal pain and why means that women can struggle to make informed judgments about proposed treatments and may be reluctant to take up hormone therapy [25] despite its life-lengthening benefits. Furthermore, severe side effects, arthralgia in particular, have been shown to be responsible for the discontinuation of hormone therapy [26-28]. A large, multi-centre study in the United States found that nearly a quarter of patients discontinued AI therapy due to musculoskeletal symptoms [28].

Musculoskeletal pain is an important issue for women as it has a negative impact on functional ability and quality-of-life [6,29]. Distress may also be caused if women are unable to differentiate between pain due to metastatic bone cancer or to treatment-aggravated musculoskeletal pain [30]. This new musculoskeletal pain is often reported as being moderate or severe and daily tasks cannot be conducted without the use of pain-killers [6,31]. Recent literature on AI-related arthralgia have identified the hands/wrists and feet/ankles as the most likely locations for joint pain and that pain in these joints has a very high impact on functional ability [6,11,19,26]. Information on musculoskeletal pain following breast cancer treatment, however, has come from retrospective analyses of large clinical trials where pain was not the focus; in addition to questions about its relationship to adjuvant therapies, the nature, duration and impact of the pain has not been established. We have therefore conducted a cohort study to establish the natural history of joint and muscle aches, pains and stiffness over time in women being treated for early breast cancer; to explore their impact on quality of life and functional ability; and to explore differences in joint and muscle aches, pains and stiffness over time between women who received different adjuvant treatments. In this paper we present the study methods and sample characteristics from baseline interviews, including hypothesised causes of musculoskeletal pain: after primary surgery but before adjuvant treatment. We describe participants’ experience of musculoskeletal pain at baseline interview and investigate the impact of surgery. We explore cancer-related quality of life, and the impact of musculoskeletal pain on this.

## Methods

The Joint Aches Cohort Study (JACS) is a prospective, multicentre cohort study studying women with non-metastatic breast cancer for one year following their primary surgery. Research ethics approval was obtained from the National Research Ethics Service, Isle of Wight, Portsmouth and South East Hampshire Research Ethics Committee, and written consent was obtained from each participant.

### Study population

Women diagnosed with primary breast cancer, either invasive carcinoma or ductal carcinoma in situ (DCIS), at one of 15 NHS hospitals across the south of England and Wales were eligible to participate in the JACS study. Recruitment to the prospective cohort commenced in January 2010 and finished in December 2011 when the target sample size of 500 patients was reached. Inclusion criteria were: a histologically confirmed diagnosis of breast cancer; no previous diagnosis of cancer (other than non-melanomatous skin cancer); at least 18 years of age; no evidence of metastatic disease; no neoadjuvant treatment; able to complete written records in English; female. Men were excluded from the study as their numbers were likely to be extremely low. A dedicated research nurse from each hospital recruited women in clinic following their primary surgery. All recruited participants were followed for a period of one year from consent.

### Sample size calculations

Recruiting over a 15-month period, as originally proposed, we estimated 100 patients with DCIS and 900 women with invasive breast cancer would present at participating clinics, with a recruitment rate of 50%. Approximately one-third of the 450 invasive breast cancer patients were estimated to be in each of the three treatment groups: 150 receiving chemotherapy only, 150 receiving hormone therapy only, and 150 receiving both chemotherapy and hormone therapy. The 50 recruited DCIS patients would form the comparison group, together with any patients with invasive cancer not receiving any adjuvant systemic therapy, as they were likely to have no further treatment. These numbers would enable us to detect a difference in prevalence of musculoskeletal pain of between 70% and 86% with 89% power.

### Data collection

Questionnaires were sent, via post, to all participants at baseline, three months, six months, nine months, and twelve months; and returned directly to the researchers using a FREEPOST envelope.

Two standardised measures of pain were used to ascertain the prevalence of musculoskeletal pain and the impact of this pain on daily life: the Nordic questionnaire, which gives data on 12 specified sites of the body [32] and the Brief Pain Inventory (BPI) [33]. To assess quality-of-life and functional status the FACT-G/FACT-B/FACT-ES [34,35] and the SF-36 [36] were used. Data on demographics, as well as co-existing conditions and potential confounders were also collected. These included presence of lymphoedema, weight, ACE inhibitor usage, use of Vitamin D and Calcium supplements. Additional questions included analgesia use; use of other medications; vitamins B6, B12, C,D and E; glucosamine; fish oil; evening primrose oil; any other complementary or herbal remedies; any other complementary therapies, including yoga, acupuncture, tai chi, aromatherapy, homeopathy and reflexology. An additional short questionnaire was sent to participants in November 2012 to obtain accurate information on current menopause status, the time since last menstrual period (less than 5 years, between 5 & 10 years, greater than 10 years), and previous use of hormone replacement therapy (HRT). Clinical information was obtained from the hospital notes including: primary surgery; adjuvant chemotherapy regimens; radiotherapy details; and the type(s) of hormone therapy prescribed. Previous diagnoses of arthritis, fibromyalgia, and menopause status were not collected as this is not accurately reported in the oncology medical notes.

### Statistical analyses

Descriptive statistics were used to compare the JACS sample with regional and national breast cancer statistics obtained from Public Health England Knowledge and Intelligence Team (South West). Further descriptive statistics were used to describe the sample, baseline pain levels, and to explore factors that might potentially confound the relationship between musculoskeletal pain and systemic primary breast cancer treatment. Forward, step-wise, multi-level logistic regression analysis was used to explain the effect of baseline joint and muscle aches and pains on quality-of-life. Further analyses explored the effect and type of surgery on baseline pain levels, specifically pain in the affected chest and upper arm regions as described by the BPI questionnaire. The data were analysed using STATA software (Version 12, SE). Data are presented with reference to the STROBE statement for cohort studies [37].

## Results

**Figure 1:**
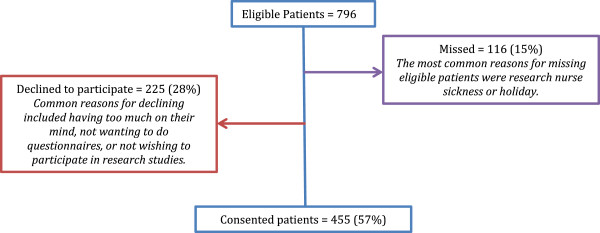
**Recruitment flowchart.** *Full eligibility data is not available for two hospitals due to long-term research nurse absence and incomplete screening logs. The numbers for these hospitals are excluded from the flowchart.

The data proved to be clustered by site, therefore seven sites that recruited fewer than 20 women were removed from all analyses, bringing the total sample down from 578 to 543 women. The clustering effect of site was included in all multi-level models. Research nurses on each site had been asked to keep records on the number of eligible patients missed and/or declined. These data were not available for two of the recruiting hospital sites, bringing the total number of eligible patients recruited to 455. The recruitment rate from the six remaining hospitals ranged from 37% to 79% of the eligible cohort. Overall, the 455 women recruited from these hospitals represent 57% of women eligible for the study. A total of 116 (15%) eligible women were missed by the recruiting nurses and a further 28% declined to participate (n = 225, Figure 1). Three patients were ineligible as it was later discovered that they had received a previous cancer diagnosis. It was originally intended to use the women with DCIS alone as a comparator to those women undergoing adjuvant therapy; however, it was found that women with DCIS were frequently given adjuvant therapy and so the data on these subjects have not been presented separately.

### Description of the sample

A total of 543 women were included in the JACS sample. Ninety-eight percent of participants returned the baseline questionnaire (n = 535) and full medical details are available for 99% of the recruited women. Age ranged from 28 to 87 years, with a mean age of 57 (Table 1). Most women were either married or in a long-term relationship (78%) and 45% were employed at least part-time. More than half (55%) were less than 60 years of age, 98% were of a White ethnic group, and 41% came from the two least deprived Index of Multiple Deprivation (IMD) quintiles.

**Table 1:** Sample demographics

	**Number**^**1**^**(%)**		**South West and Oxford Cancer Registry Areas, 2009–2011**^**2**^**(%)**	**England, 2009–2010 (%)**^**2**^
**Age group**				
<40	31 (6%)	285 (55%)	10,535 (40%)	33,885 (41%)
40-49	100 (19%)			
50-59	154 (30%)			
60-69	170 (33%)	237 (45%)	15,653 (60%)	47,901 (59%)
≥70	67 (13%)			
**Ethnicity**				
White	510 (98%)		Information not available	78,075 (95%)
Non-white (Asian/Chinese/Mixed)	9 (2%)			3,711 (5%)
**Marital status**				
Married/Long-term relationship	412 (78%)		Information not available	Information not available
Single/Divorced/Widowed	113 (22%)			
**Socio-economic group**				
Employers & managers	57 (11%)		Information not available	Information not available
Professionals	64 (12%)			
Intermediate non-manual	137 (26%)			
Junior non-manual	123 (23%)			
Manual (skilled & unskilled)	40 (8%)			
Other	45 (8%)			
Not known	67 (13%)			
**Current employment status**				
Employed (full-time)	131 (25%)		Information not available	Information not available
Employed (part-time)	122 (23%)			
Homemaker	48 (9%)			
Unemployed	16 (3%)			
Retired	182 (35%)			
Other	27 (5%)			
**Index of multiple deprivation (IMD) quintiles**^**3**^				
1 (Most deprived)	46 (11%)		7,373 (28%)	Information not available
2	105 (25%)		7,091 (27%)	
3	105 (25%)		6,071 (23%)	
4	105 (25%)		4,118 (16%)	
5 (Least deprived)	70 (16%)		1,535 (6%)	

^1^Where numbers do not equal 543 missing data are present.

^2^Data supplied by Public Health England Knowledge and Intelligence Team (South West).

^3^IMD Quintile data are only comparable within England – patients recruited in Wales were excluded from these calculations (n = 29).

All the women in the cohort had primary breast cancer surgery. Wide local excision (WLE) was the most common surgery (67%) and 33% of women had a single or double mastectomy (Mx). Sentinel lymph node biopsies (SLNB) were undertaken in 56% of women and 30% had axillary node clearance (ANC) (Table 2). At the time of surgery, 45% of the sample was scheduled to have adjuvant chemotherapy, of whom 43% were scheduled to have taxane-containing chemotherapy (Table 2). Most women were scheduled to commence adjuvant hormone therapy; 28% of the women were scheduled to take an initial AI therapy while 43% were scheduled to start tamoxifen. The hormone therapy prescribed for post-menopausal women varied considerably by site: for example, one hospital prescribed tamoxifen in preference to AIs to 78% of post-menopausal women whilst others prescribed AIs as a first-line treatment to 75% of women.

**Table 2:** Surgery and planned breast cancer treatment

	**Number (%)**
**Surgery type**	
Mastectomy	15 (3%)
Mastectomy + sentinel node biopsy	69 (13%)
Mastectomy + axillary clearance	95 (18%)
Wide local excision (WLE)	63 (12%)
WLE + sentinel node biopsy	230 (43%)
WLE + axillary clearance	68 (13%)
**Planned cancer treatment**	
No further systemic treatment	82 (16%)
Aromatase inhibitor only	95 (18%)
Tamoxifen only	117 (22%)
Chemotherapy only	72 (14%)
*of which taxane chemotherapy – 37 (51%)*	
Aromatase inhibitor + chemotherapy	52 (10%)
*of which taxane chemotherapy – 16 (31%)*	
Tamoxifen + chemotherapy	107 (20%)
*of which taxane chemotherapy – 51 (48%)*	

Only 37% of women reported no other long-term health conditions (n = 195). Hypertension was reported by 26% of women. Over 50% of the sample (n = 245) was classed as being overweight or obese (BMI > 25) (Table 3). The majority of the women were post-menopausal (64%) and 33% reported previous HRT use, which may related to the number reporting a hysterectomy (n = 85). Nearly a quarter of our sample reported a diagnosis of an arthritis, joint or muscle related condition (rheumatoid arthritis, osteoarthritis, other arthritis, or fibromyalgia) (Table 3).

**Table 3:** Medical characteristics of the sample

	**Number (%)**
**BMI**	
Underweight (BMI <18)	7 (1%)
Health weight (BMI 19–25)	235 (48%)
Overweight (BMI 26–29)	120 (25%)
Obese (BMI ≥30)	125 (26%)
**Menopause status**^**1**^	
Pre-menopausal	45 (10%)
Peri-menopausal	56 (13%)
Post-menopausal	277 (64%)
Patient unsure	53 (12%)
**Time since last menstrual period**^**2**^	
<5 years	76 (23%)
5-10 years	72 (22%)
>10 years	185 (56%)
**Previous HRT use**	
Yes	144 (33%)
**Musculoskeletal diagnosis at baseline**^**3**^	
Osteoarthritis	73 (14%)
Rheumatoid arthritis	16 (3%)
Fibromyalgia	7 (1%)
Other arthritis	33 (6%)
**Taking other medications known to cause joint pain**	
*(ace inhibitors, proton pump inhibitors, quinolones, tibolones, zoledronic acid, bisphosphonates, angiotensin-II receptor antagonists, etc.)*	
Yes	105 (20%)
**Osteo-enhancing supplements**^**3**^	
Vitamin D	16 (3%)
Calcium	45 (8%)
Glucosamine	55 (10%)

^1^Menopause status as defined by the patient.

^2^Does not include pre-menopausal women or women with unknown status.

^3^Percentages are % of total cohort and will not sum to 100%.

Information was also collected on other causes of musculoskeletal pain, to enable their effects to be controlled for in later analysis when exploring the effects of adjuvant treatment. These included weight, a history of joint pain or arthritis, other long term conditions and some medications (including ACE inhibitors, gastric acid reducers, quinolones, tibolones, and zoledronic acid) [38] have all been monitored in this sample (Table 3). Twenty percent of women in this study were on medications known to cause arthralgia (n = 105). Menopause status and the time since their last menstrual period were monitored due to the believed role of oestrogen deprivation in the development of musculoskeletal pain. Nearly 80% of the JACS cohort were peri or post-menopausal with only 23% of women reporting their last menstrual period within the previous five years (Table 3). Some studies have also linked Vitamin D and other seasonal effects as having a significant role in joint pain development in women with breast cancer [31,39-41]. Only 9% of the cohort reported taking an osteo-enhancing supplement (defined as Vitamin D, Calcium, or Glucosamine) at baseline; fewer than 20 women reported a daily Vitamin D supplement.

### Pain prevalence

The Nordic Musculoskeletal questionnaire and the BPI both use broad definitions of pain; the Nordic questionnaire includes any ‘ache, pain, stiffness, discomfort or numbness’ and the BPI asks: ‘most of us have had pain from time to time (such as minor headaches, sprains and toothache). Have you had pain other than these everyday kinds of pain **today**’. We, therefore, added to the Nordic questionnaire six yes/no questions as to whether the respondent had any joint pain/joint aches/joint stiffness/muscle aches/muscle pain/muscle stiffness in the last seven days. These questions were compared to the initial BPI pain question: 53% of the sample reported pain in both instruments but a further 47% identified having musculoskeletal pain in our additional questions that was not recorded using the BPI questionnaire. This difference may be due to the time span of the questions: our additional questions ask about musculoskeletal pain in the previous seven days whilst the BPI questionnaire focusses on pain in the current 24 hour period.

**Figure 2:**
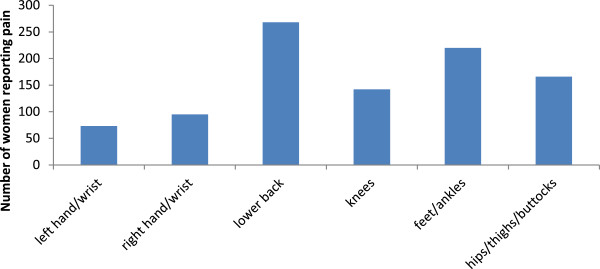
Reported pain in previous 12 months as identified in the Nordic Musculoskeletal questionnaire.

Nearly 70% of women report musculoskeletal pain, including joint or muscle pain, aches, or stiffness, in the previous seven days in the questions added to the Nordic questionnaire (n = 391; 69%; 95% CI 65%, 73%), while only 28% (n = 146; 95% CI 24%, 32%) report joint aches/pains/stiffness. The BPI questionnaire asks participants to identify the location of the greatest source of pain within the previous 24 hours, as well as to identify all other pain locations [33]. Most of the women completing the baseline questionnaire did not specify a greatest pain location, but the lower back was identified as the most frequently cited ‘area that hurts the most’ (13%). On the Nordic musculoskeletal questionnaire, the most commonly reported pain sites over the previous 12 months included: lower back pain (50%); pain in one or both knees (41%); and pain in their hips/thighs/buttocks (31%, Figure 2). In the 12 months preceding primary breast cancer surgery, 26% of women report right wrist or hand pain and 20% report left hand/wrist pain on the Nordic Musculoskeletal questionnaire; fewer than 5 women identified these sites as the location of greatest pain on the BPI questionnaire.

Women were recruited to the JACS study immediately following primary breast cancer surgery, which offers an opportunity to examine how pain changes in the weeks following surgery. The following analyses excluded women who completed the baseline questionnaire prior to surgery (n = 21), more than 90 days after surgery (n = 11), and those with no identified surgery date (n = 21). In the remaining 491 women, 70% reported joint or muscle aches/pains/stiffness in the previous 7 days. One in three (35%) of women reported pain in the shoulder, chest wall, or underarm on the surgical side. This is the type of pain than can likely be attributed to surgical intervention (95% CI 29%, 41%). More women who had a mastectomy reported this type of pain (48%) compared with those receiving a WLE (27%, p = 0.002). The women most likely to be reporting surgical pain had surgery in the previous 30 days (48%) but there was no difference in pain levels between women 31–60 days post-surgery and those 61–90 days post-surgery. Over-the-counter (OTC) pain medication usage also varied over time (p = 0.002) with 49% of women with surgery in the previous 30 days reporting OTC pain relief usage, 33% of those with surgery in the previous 31–60 days, and 48% with surgery in the previous 61–90 days.

### Relationship between musculoskeletal pain and quality of life

Baseline pain scores from the BPI questionnaire, SF-36 quality-of-life domain scores, and the FACIT scores are presented in Table 4; these scores are further subdivided by those that responded ‘yes’ to any of the questions added to the Nordic Musculoskeletal questionnaire (any of the following in the previous seven days: joint pain; joint aches; joint stiffness; muscle aches; muscle pain; or muscle stiffness). Baseline musculoskeletal pain, as defined from our additional questions, was associated with significantly worse quality of life in all domains of the SF-36 and all the FACIT domains, other than social and emotional well-being.

**Table 4:** Pain and quality of life

	**Total cohort**	Musculoskeletal pain*	**No musculoskeletal pain**	**t-test pain vs no pain**
	**mean (SD)**	**mean (SD)**	**mean (SD)**	
**Brief pain inventory (BPI) scores**				
Pain severity	3.44 (1.79)	3.58 (1.79)	2.64 (1.60)	p = 0.006
Pain interference	2.87 (2.31)	3.09 (2.33)	1.55 (1.62)	p = 0.05
**SF-36 domains**				
Physical functioning	74.00 (25.80)	67.82 (27.10)	87.20 (16.61)	p < 0.001
Role - physical	37.93 (44.21)	31.92 (42.35)	50.75 (45.54)	p < 0.001
Bodily pain	60.68 (26.30)	55.01 (25.36)	72.33 (24.32)	p < 0.001
General health	69.44 (19.00)	66.25 (19.88)	76.21 (14.89)	p < 0.001
Vitality	53.53 (20.60)	49.53 (20.56)	61.99 (17.91)	p < 0.001
Social functioning	64.22 (25.88)	60.31 (25.71)	72.44 (24.65)	p < 0.001
Role - emotional	61.17 (43.54)	56.82 (43.92)	69.53 (41.44)	p = 0.003
Mental health	69.35 (16.96)	67.93 (16.92)	72.34 (16.56)	p = 0.006
**FACT scores**				
Physical well-being	21.69 (5.08)	20.79 (5.27)	23.68 (4.02)	p < 0.001
Social well-being	24.40 (4.48)	24.30 (4.46)	24.54 (4.35)	p = 0.98
Emotional well-being	18.50 (4.14)	18.33 (4.12)	18.89 (4.14)	p = 0.17
Functional well-being	18.94 (6.35)	18.26 (6.33)	20.50 (6.13)	p < 0.001
FACT-G total score	81.25 (15.07)	79.31 (15.45)	85.51 (13.38)	p < 0.001
FACT-B total score	104.27 (19.42)	101.72 (20.12)	109.83 (16.77)	p < 0.001
FACT-ES total score	166.84 (25.69)	162.30 (26.53)	176.58 (20.97)	p < 0.001

*Musculoskeletal pain as defined by a ‘yes’ answer to at least one of our six questions added to the Nordic Musculoskeletal questionnaire. The Brief Pain Inventory describes all types of pain in the previous 24 hours; both measures are on a scale of 0–10, with 10 being the most severe pain or pain that most interferes with daily life. The SF-36 domains are on a scale of 0–100, with a high score indicating a more favourable health state. The FACIT physical, social, and functional well-being subscales are on a scale of 0–28; the emotional well-being sub-scale ranges from 0–24; the FACT-G total score ranges from 0–108, FACT-B 0–144, FACT-ES 0–220; higher scores represent a better QoL.

A further multi-level linear regression analysis looking at the FACT-B total score and baseline pain was conducted; the final model included 480 women (Table 5). The results were adjusted for site (clustering), age, a diagnosis of fibromyalgia, previous diagnosis of depression, axillary node clearance vs sentinel lymph node biopsy, opioid painkiller usage, and OTC painkiller usage. Time since surgery and mastectomy vs wide local excision did not have a statistically significant effect. The FACT-B total score was reduced by 5 points (95% CI -8.95, -1.76) in women with musculoskeletal pain, as defined by our addition to the Nordic Musculokeletal questionnaire, after adjusting for all the confounding factors (p = 0.0038).

**Table 5:** Linear regression model FACT-B total score and musculoskeletal pain

	**Change in mean FACT-B score**	**95% CI**	**p-value**^**1**^
Baseline constant	106.01	96.62, 115.41	p < 0.001
Report musculoskeletal pain	-5.05	-8.78, -1.33	p = 0.008
Age	+0.19	+0.03, +0.34	p = 0.017
History of fibromyalgia	-16.95	-31.40, -2.49	p = 0.022
History of depression	-8.44	-13.36, -3.52	p = 0.001
Surgery: sentinel node biopsy	-9.49	-13.30, -5.68	p < 0.001
Surgery: axillary clearance	-4.82	-9.62, -0.03	p = 0.049
Current opioid painkiller use	-9.01	-13.65, -4.36	p < 0.001
Current OTC painkiller use	-4.30	-7.73, -0.87	p = 0.014
Hospital recruitment site	-0.09	-0.59, +0.41	p = 0.721

^1^Overall Likelihood ratio estimate for the model, p = 0.0038.

## Discussion

This paper presents the first findings from a large prospective multi-centre cohort study to establish the natural history of joint and muscle aches, pains and stiffness over time in women being treated for early breast cancer; to explore their impact on quality of life and functional ability; and to explore differences in joint and muscle aches, pains and stiffness over time between women who received different adjuvant treatments. It demonstrates the success of the study in recruiting over 500 women after primary breast cancer treatment and before adjuvant cancer treatment, describes methods and sample characteristics, and presents findings on the prevalence of musculoskeletal pain, the impact on it of breast cancer surgery, and the effect of these pains on quality of life.

Nearly three quarters of women (69%) reported joint or muscle pain, aches or stiffness after primary cancer surgery but before adjuvant therapy. This is a similar proportion to that reported in our group’s previous cross-sectional research (62%) [12]. When only questions on joint pains, aches or stiffness were included in the current study this dropped to 28%. We used questions specific to musculoskeletal pain, aches and stiffness which produced higher musculoskeletal pain prevalence figures than the Brief Pain Inventory, used in other studies [20], which investigates general pain experience rather than focusing on specific sites. By exploring the sites and prevalence of pain attributed to primary breast cancer surgery we were able to establish that this does not account for the high rates of musculoskeletal pain reports in women with breast cancer. Validation of our pain data is, however, provided by the fact that the pain reports after surgery are as might be expected with, for example, more pain in the arm and shoulder of the affected side, less pain with time, and more pain after a mastectomy than a WLE.

Our results show the importance of understanding more about the natural history, aetiology and treatment of musculoskeletal pain in women with breast cancer: even in the midst of the emotional turmoil of breast cancer diagnosis, recent surgery and consideration of adjuvant therapies, musculoskeletal pain still had a statistically significant negative association with quality of life. Women who reported this pain reported lower physical functioning, general health, vitality and mental health on the SF-36, a general measure of health-related quality of life; they reported more impact on their physical and emotional roles and had more bodily pain. This was also reflected on the FACT-G and B, cancer-focused quality-of-life scales, where women with these pains again reported lower quality of life. We used multi-level, multi-variate analysis to control for clustering and the effect of confounding variables such as time since surgery, use of analgesia, depression and age: the negative impact of these pains on quality-of-life remains. Further findings from our cohort study will help ensure these symptoms can be effectively addressed and their impact on quality of life alleviated.

Our study has limitations. Our sample are younger than would be expected from regional and national data, with 55% less than 60 years of age compared with 40% of breast cancer diagnoses in the South West and Oxford Cancer Registry areas and 41% of breast cancer diagnoses in England. They are also from more affluent backgrounds as we have oversampled from the two least deprived Index of Multiple Deprivation (IMD) quintiles. These are not uncommon biases in research samples, and, importantly we did not exclude older women, with women aged 70 or above comprising 13% of our sample. We were able to recruit 57% of our target population, with other eligible women either being missed by research nurses in busy breast cancer clinics or declining to take part. This may have introduced other, unknown, biases into the study.

## Conclusions

In conclusion, musculoskeletal pain is a common problem following primary breast cancer treatment, but there is little information on the natural history of this symptom and its impact on women’s lives over time. This cohort study of 543 women has been established to explore the natural history of joint and muscle aches, pains and stiffness, their impact on quality of life and to begin to explore how these differ between different treatment groups. In this paper we demonstrate the successful establishment of the cohort and describe study methods and sample characteristics. We report the prevalence of musculoskeletal pain and joint pain in women after primary breast cancer and before adjuvant therapy, and demonstrate its negative association with quality of life. This highlights the importance of a better understanding of these symptoms and their impact on the lives of women with primary breast cancer so that healthcare professionals are better equipped to support patients and to provide accurate information to inform treatment decisions. Further papers from this study will address these issues.

## Competing interests

In the last five years, Dr Peter Simmonds has received honoraria from Novartis for several presentations on management of gastrointestinal stromal tumours and honoraria from Novartis, Pfizer, and Roche for attending advisory boards on letrozole, sunitinib, and trastuzumab and has been supported to attend a number of conferences funded by Novartis and Pfizer. Dr Deborah Fenlon has received an honorarium from Roche to present on hormone treatment in breast cancer. All other authors have no conflicts of interest.

## Authors’ contributions

DF conceived of the study, led its design and obtained funding with JAH, managed the establishment and co-ordination of the cohort, and helped to draft the manuscript. CP participated in the coordination of the study, performed the statistical analyses, and helped to draft the manuscript. PS and JC participated in the design of the study and guided analyses. JAH led the design of the study, obtained funding with DF, led the establishment of the cohort, and helped to draft the manuscript. All authors read and approved the final manuscript.

## Pre-publication history

The pre-publication history for this paper can be accessed here:

http://www.biomedcentral.com/1471-2407/14/467/prepub
